# Chronic Effects of *Fusarium* Mycotoxins in Rations with or without Increased Concentrate Proportion on the Insulin Sensitivity in Lactating Dairy Cows

**DOI:** 10.3390/toxins10050188

**Published:** 2018-05-08

**Authors:** Asako Kinoshita, Christina Keese, Ulrich Meyer, Alexander Starke, Christine Wrenzycki, Sven Dänicke, Jürgen Rehage

**Affiliations:** 1 Clinic for Cattle, University of Veterinary Medicine Hannover, Bischofsholer Damm 15, 30173 Hannover, Germany; asakokinoshita@googlemail.com; 2 Institute of Animal Nutrition, Friedrich-Loeffler-Institute (FLI), Federal Research Institute for Animal Health, Bundesallee 37, 38116 Braunschweig, Germany; Christina.Keese@gmail.com (C.K.); Ulrich.Meyer@fli.de (U.M.); Sven.Daenicke@fli.de (S.D.); 3 Clinic for Ruminants and Swine, Faculty of Veterinary Medicine, University Leipzig, An den Tierkliniken 11, 04103 Leipzig, Germany; alexander.starke@vetmed.uni-leipzig.de; 4 Faculty of Veterinary Medicine, Justus-Liebig-University Giessen, Clinic for Veterinary Obstetrics, Gynecology and Andrology, Chair for Molecular Reproductive Medicine, Frankfurter Straße 106, 35392 Giessen, Germany; Christine.Wrenzycki@vetmed.uni-giessen.de

**Keywords:** deoxynivalenol, dairy cow, insulin sensitivity, energy metabolism

## Abstract

The objective of this study was to investigate the effect of long-term exposure to a *Fusarium* toxin deoxynivalenol (DON, 5 mg/kg DM) on the energy metabolism in lactating cows fed diets with different amounts of concentrate. In Period 1 27 German Holstein cows were assigned to two groups and fed a control or mycotoxin-contaminated diet with 50% concentrate for 11 weeks. In Period 2 each group was further divided and fed either a diet containing 30% or 60% concentrate for 16 weeks. Blood samples were collected in week 0, 4, 8, 15, 21, and 27 for calculation of the Revised Quantitative Insulin Sensitivity Check Index and biopsy samples of skeletal muscle and the liver in w 0, 15, and 27 for analysis by real-time RT-qPCR. The DON-fed groups presented lower insulin sensitivities than controls at week 27. Concomitantly, muscular mRNA expression of insulin receptors and hepatic mRNA expression of glucose transporter 2 and key enzymes for gluconeogenesis and fatty acid metabolism were lower in DON-fed cows compared to the control. The study revealed no consistent evidence that DON effects were modified by dietary concentrate levels. In conclusion, long-term dietary DON intake appears to have mild effects on energy metabolism in lactating dairy cows.

## 1. Introduction

Deoxynivalenol (DON) is one of the mycotoxins that are most prevalently detected in feedstuffs [1]. Among livestock, cattle are less sensitive to DON intoxication due to the detoxification mechanisms in the rumen [2]. Most of ingested dietary DON is degraded during the process of ruminal microbial fermentation to the by far less toxic de-epoxy-deoxynivalenol DOM-1 [3,4]. The ruminal fermentation pattern, and thereby possibly the ruminal DON degradation capacity, depends on the concentrate proportion of the diet [5,6]. In high-yielding dairy cows, the capacity for ruminal detoxification may be restricted by additional metabolic burdens for ruminal microbes, such as concentrate-rich diet, or by faster rumen turnover rates [7]. Therefore, varying the dietary proportion of concentrate might influence the detoxification of DON. In dairy cattle, ingestion of a DON-contaminated diet affected ruminal fatty acid composition [6] and ruminal protein utilization [3]. Regarding the effects on the liver, no evidence was found for the effects of DON on the integrity and morphology of hepatocytes as assessed by plasma activity of hepatic enzymes and by histopathological evaluation in dairy cattle [8,9]. On the other hand, immunosuppressive effects of DON have been observed in cattle in vitro [10,11,12] and in vivo [13,14].

Recently the toxicological relevance, the pathophysiological effects, and their underlying mechanism of DON were reviewed by Pestka [15] and Mareska [16] in detail. Their “ribotoxic effects” mainly cause the cytotoxic effects of DON and other trichothesenes, where these toxins bind to ribosomes of eukaryotic cells, leading to the inhibition of the translation and the activation of mitogen-activated protein kinases (MAPK). Through the activation of MAPK the transcription factors (e.g., NF-κB) are modulated and the expression of downstream genes are altered. This process seems to be the main mechanism of DON effects on the immune system [17,18]. The immunosuppressive effects of DON observed in cattle might indicate the existence of similar mechanisms of DON action in this species.

A reduced insulin sensitivity was associated with metabolic disorders of dairy cows in the periparturient period, which eventually could lead to disturbed function of the reproduction system and/or immune system [19,20,21]. The revised insulin sensitivity check index (RQUICKI), determined from the basal concentration of insulin, glucose, and non-esterified fatty acid (NEFA), has been used as a surrogate indicator to assess the systemic insulin sensitivity in cattle [19,21]. The systemic insulin sensitivity is dependent on the insulin-sensitive tissues, above all, liver, muscle, and adipose tissue [20]. In the muscle insulin promotes the glucose uptake by translocating the insulin-dependent glucose transporter 4 (GLUT4) from intracellular stored vesicles to the plasma membrane and thereby increases the utilization of glucose. While most of the whole body insulin-dependent glucose uptake takes place in the muscle, in adipose tissue the antilipolytic function of insulin is important. In the liver, insulin suppresses gluconeogenesis and fatty acid oxidation and promotes fatty acid esterification and synthesis of fatty acid [20,22].

Both DON contamination of feedstuffs and concentrate-rich diets are common in the modern dairy production system. Thus, it is of interest whether long-term ingestion of a DON-contaminated diet is associated with reduced insulin sensitivity and with altered expression of genes involved in energy metabolism in liver and muscle, and whether these effects of DON are enhanced by the increased concentrate proportion in the diet.

Therefore, a feeding trial for 27 weeks was conducted using high-yielding German Holstein cows during mid- to late lactation. The experiment was divided into two periods. During the first eleven weeks in Period 1 cows were allocated either to control (CON) or mycotoxin-fed (MYC) groups, and fed diets with almost identical composition except for the dietary DON content (50%:50% concentrate-to-forage-proportion on a dry matter basis). In the following 16 weeks in Period 2 each CON and MYC group was divided, fed on low (CON30 and MYC30) or high (CON60 and MYC60) dietary concentrate levels (30%:70% vs. 60%:40% concentrate-to-forage-proportion on a dry matter basis). The blood metabolites as indicators for the systemic insulin sensitivity and transcription levels of glucose transporters and insulin receptors in muscle and liver, and those of key enzymes involved in gluconeogenesis and fatty acid metabolism in the liver were monitored.

## 2. Results

### 2.1. Dry Matter Intake, Body Weight, Milk Production Performance, and Mycotoxin Intake

The effect of DON and the concentrate levels on dry matter intake (DMI), body weight (BW), milk production performance, and mycotoxin intake have been published by Keese et al. [23]. In short, the average DON intake was significantly higher in the cows fed a mycotoxin-contaminated diet compared to the control in Period 1. In Period 2, the DON intake was similar to those in Period 1 in the control groups in both low (CON30) and high (CON60) concentrate levels. In the cows fed a mycotoxin-contaminated diet, the DON intake was higher in the cows fed the high-concentrate level (MYC60, 153.8 ± 1.2 µg/kg BW) than the cows fed the low-concentrate level (MYC30, 122.6 ± 1.1 µg/kg BW) (data shown in mean ± standard error). In Period 1 the mycotoxin-contaminated diet led to a higher DMI (16.9 ± 0.1 vs. 19 ± 0.1 kg/d for CON vs. MYC), body weight (573 ± 2.9 vs. 594 ± 3.7 kg for CON vs. MYC), and milk yield (28.0 ± 0.4 vs. 31.0 ± 0.5 kg/d for CON vs. MYC). In Period 2, the DMI was generally higher in cows fed the high-concentrate level (18.4 ± 0.4 and 19.2 ± 0.4 kg/d for CON60 and MYC60) than the others (CON30 and MYC30) and, for the low-concentrate level, it was significantly higher in MYC30 (16.8 ± 0.4 kg/d) than in CON30 (15.3 ± 0.4 kg/d). No effect of the diet was detected on the BW in Period 2. Milk yield was significantly higher in cows fed a mycotoxin-contaminated diet than in control cows the high-concentrate level (24.9 ± 1.4 vs. 30.0 ± 1.4 kg for CON60 vs. MYC60), while there was no difference in milk yield in cows fed the low-concentrate level (23.4 ± 1.3 vs. 26.9 ± 1.3 kg/d for CON30 vs. MYC30).

### 2.2. Blood Metabolites and Insulin Sensitivity

**Figure 1:**
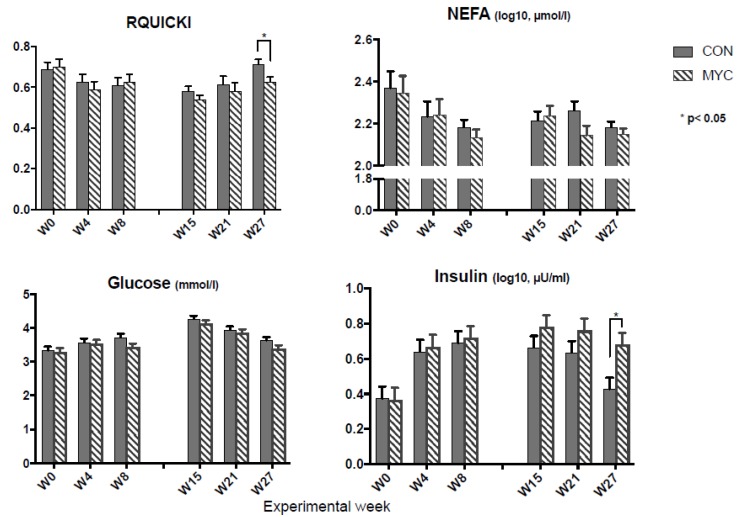
Effects of mycotoxins on RQUICKI and serum concentration of NEFA, glucose, and insulin in Period 1 (W0–W8) and Period 2 (W15–W17). The data are shown as mean values pooled for the factor of mycotoxin × week + standard error of means. RQUICKI: revised quantitative insulin sensitivity check index; NEFA: non esterified fatty acid; CON: control group (*n* = 14); MYC: mycotoxin group (*n* = 13).

**Figure 2:**
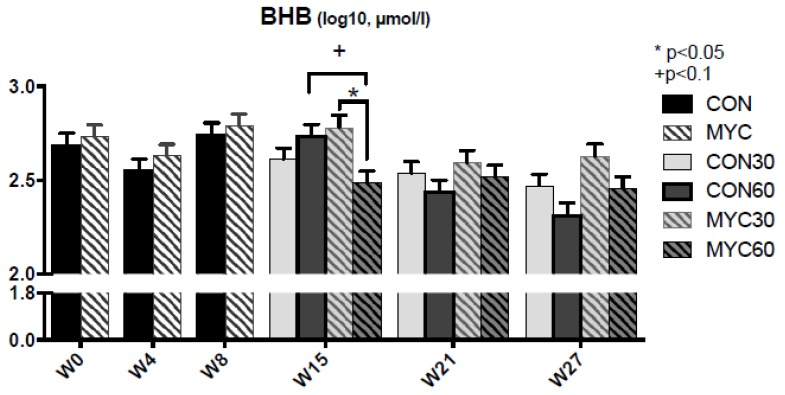
Effects of mycotoxins and concentrate proportion on serum concentration of BHB (beta-hydroxybutyrate) in Period 1 (W0–W8) and Period 2 (W15–W17). The data are shown as mean values for each experimental group and sampling week + standard error of means. CON: control group; MYC: mycotoxin group; CON30 or CON60: control group with 30% or 60% concentrate proportion in the diet; MYC30 or MYC60: mycotoxin group with 30% or 60% concentrate proportion in the diet.

The results of the analysis of serum concentration of glucose, insulin, NEFA, beta-hydroxybutyrate (BHB), and RQUICKI are summarized in Table 1. The medians (ranges) of original data of insulin, NEFA, and BHB were 3.78 (1.0–16.9) µU/mL, 157 (29.11–1095) µmol/L, and 0.48 (0.17–2) mmol/L in Period 1 and 4.5 (0.432–22.5) µU/mL, 159 (60.5–647) µmol/L, and 0.36 (0.14–0.97) mmol/L in Period 2, respectively. In Period 1 no mycotoxin effect was detected, while time-related changes were significant in all the analyzed variables, except for BHB (Table 1). In Period 2 significant time effects were found for all studied variables, except NEFA (Table 1, Figure 1 and Figure 2). Results of insulin and RQUICKI assessment revealed significant mycotoxin effects with generally greater insulin and lower RQUICKI results in cows of MYC groups compared to controls (Table 1, Figure 1). An interaction effect for week × mycotoxin was found for BHB concentrations. The concentrate level in the diet affected NEFA, BHB, and insulin significantly (Table 1) and week × concentrate effect was seen for NEFA (Table 1). Only for BHB was an interaction effect of week × concentrate × mycotoxin detected (Table 1, Figure 2). At Week 15 cows of the MYC60 group presented lower mean BHB concentration than cows of CON60 and MYC30 groups (Figure 2).

### 2.3. Muscular mRNA Expression of Glucose Transporters and Insulin Receptors

**Figure 3:**
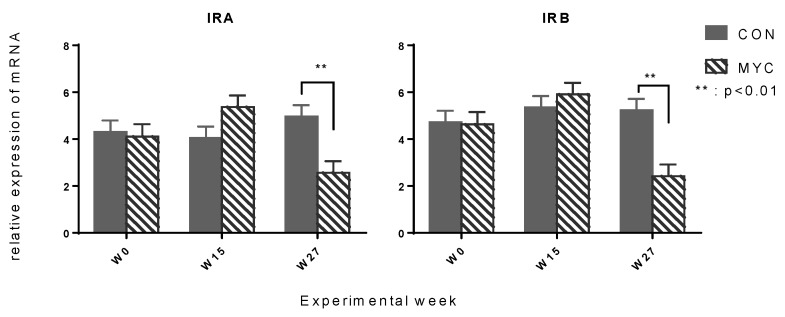
Effects of mycotoxins on muscular mRNA expression of insulin receptor A and B (IRA and IRB). The data are shown as mean values pooled for the factor of mycotoxin × week + standard error of means. CON: control group (*n* = 10); MYC: mycotoxin group (*n* = 10).

The results of the analysis of muscular mRNA expression are presented in Table 2. The cows fed a mycotoxin-contaminated diet presented a significantly lower relative amount of mRNA of insulin receptors (isotypes IRA and IRB) at Week 27 (Figure 3). A mycotoxin × concentrate level × week interaction effect was found in SLC2A4. High concentrate level in the diet led to an upregulation of SLC2A4 in control groups in Week 27, while it led to downregulation in the MYC groups. A significant difference was detected between CON60-MYC60. Similar pattern of differences in mRNA amount among the experimental groups was observed also in IRA and IRB, reflecting the relatively lower *p*-values < 0.1 for the mycotoxin × concentrate level × week interaction effect in the analysis of variances. The mRNA amount of SLC2A1 was higher in MYC groups affected by the experimental week and mycotoxin, but no interaction of both factors was found, indicating the observed mycotoxin effect was probably not due to the dietary intervention.

### 2.4. Hepatic mRNA Expression of Enzymes Involved in Gluconeogenesis and Oxidation and Esterification of Fatty Acid

**Figure 4:**
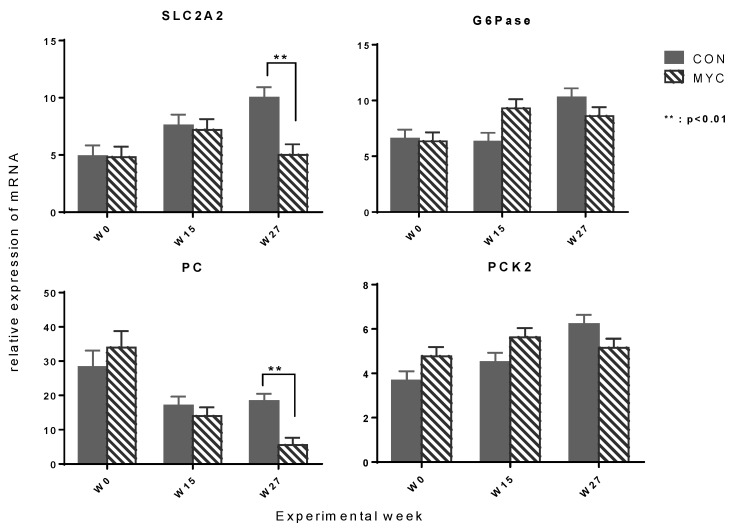
Effects of mycotoxins on hepatic mRNA expression of glucose transporter 2 (SLC2A2) and key enzymes for gluconeogenesis (G6Pase, PC, PCK2). The data are shown as mean values pooled for the factor of mycotoxin × week + standard error of means. The demonstrated genes were affected by mycotoxin × week interaction effect with *p* < 0.05 (SLC2A2, PC, and PCK2) or *p* = 0.051 (G6Pase) in the analysis of variances. CON: control group (*n* = 10); MYC: mycotoxin group (*n* = 10). G6Pase: glucose-6-phosphatase; PC: pyruvate carboxylase; PCK2: phosphoenolpyruvate carboxykinase 2.

**Figure 5:**
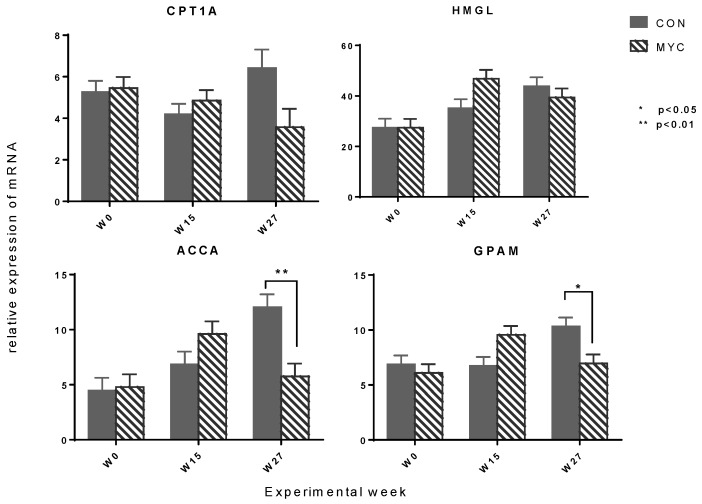
Effects of mycotoxins on hepatic mRNA expression of key enzymes for fatty acid metabolism. The data are shown as mean values pooled for the factor of mycotoxin × week + standard error of means. CON: control group (*n* = 10); MYC: mycotoxin group (*n* = 10). The demonstrated genes were affected by mycotoxin × week interaction effect with *p* < 0.05 (CPT1A, ACCA, and GPAM) or *p* = 0.07 (HMGL) in the analysis of variances. CPT1A: carnitine palmitoyltransferase 1A; HMGL: 3-hydroxymethyl-3-methylglutaryl-CoA lyase; ACCA: acetyl-CoA carboxylase; GPAM: mitochondrial glycerol phosphate acyltransferase.

The results of the analysis of the hepatic mRNA expression are presented in Table 3, Table 4 and Table 5. A mycotoxin × week interaction effect was found to be significant in glucose transporter 2 (SLC2A2), pyruvate carboxylase (PC), phosphoenolpyruvate carboxykinase 2 (PCK2) (Figure 4), carnitine palmitoyl transferase 1A (CPT1A), acetyl-CoA carboxylase A (ACCA), and mitochondrial glycerol phosphate acyltransferase (GPAM) (Figure 5). In all of these genes, the relative amount of the mRNA was lower in cows fed the mycotoxin-contaminated diet (MYC30 and MYC60) compared to those in control groups (CON30 and CN60) at Week 27. The difference of mean values was significant in SLC2A2, PC, ACCA, and GPAM. Similar pattern of mRNA expression among the experimental groups was also observed in glucose-6-phosphatase (G6Pase) and 3-hydroxymethyl-3-methylglutaryl-CoA lyase (HMGL) with *p*-values < 0.1 for the mycotoxin × week interaction effect. A concentrate level × week interaction was found in gluconeogenetic enzymes G6Pase, PCK1, and propionyl-CoA carboxylase (PCCA) and in ketogenic enzymes CPT1A, 3-hydroxy-3-methylglutaryl-CoA synthase 2 (HMGS2), and HMGL with greater amount of mRNA in cows fed diets with a low concentrate level than those fed diets with a high concentrate level at Week 15 and/or 27. The differences of mean values were significant in Week 15 in PCK1 (group mean ± standard error for 30%- vs. 60%-group: 17.76 ± 1.66 vs. 10.38 ± 1.66, *n* = 10/group, *p* = 0.003) and at Week 27 in G6Pase (10.75 ± 0.80 vs. 8.13 ± 0.81, *p* = 0.0275). No mycotoxin × concentrate level × week interaction effect was found.

## 3. Discussion

### 3.1. Study Design, Dose of DON, and Effect of Co-Contamination by Zearalenone

The design in the current study allowed testing the long-term mycotoxin effects over a 27 week study period, while the hypothesized interaction of mycotoxin and concentrate was tested only in Period 2. Since adaptation of ruminal fermentation and metabolism to new diets is assumed to take about three to four weeks [5], it appeared to be justified to consider a dietary concentrate effect in the statistical evaluation of results of samples taken in Week 15 which was three weeks after the diets were changed.

In MYC groups, the DON contamination in the diet was at about 5.5 mg/kg and 4.5 mg/kg dry matter in Period 1 and 2, respectively. These levels were close to the tolerance limit at 5.7 mg/kg dry matter set by European Commission [24]. The dietary DON content in control cows was about 0.6 mg/kg dry matter in both periods (Table 6) and, accordingly, the average DON intake was significantly higher in cows in the MYC group than control cows in both Period 1 and Period 2. In contrast, the concentrations of the dietary zearalenone (ZEN), mycotoxin produced by *Fusarium* spp., were, on average, 113 µg/kg and 73 µg/kg dry matter in Period 1 and 2, respectively in the MYC groups, and in the CON groups they were, on average, 53 µg/kg and 35 µg/kg dry matter in Period 1 and 2, respectively. These dietary ZEN contents are far below the tolerance limit of 0.57 mg/kg dry matter set by the European Commission, 2006 [24]. Thus, DON was fed to MYC cows in practically relevant amounts, and assumed as the tested dominating mycotoxin in this feeding trial.

### 3.2. Blood Analysis and RQUICKI

In Period 1 DON exposure revealed no significant effects on insulin and other blood metabolites as observed in previous studies with comparable study terms and DON intakes [8,13,25,26,27,28]. Accordingly, no mycotoxin effect was found in RQUICKI. In Period 2 mycotoxin-contaminated diets led to significantly higher serum insulin concentration in Week 27 and lower RQUICKI levels.

RQUICKI, which is based on insulin, NEFA, and glucose blood concentrations is widely used in human medicine as an indicator for insulin sensitivity of peripheral tissues, and is also suggested for application in dairy cows [19,21]. The mycotoxin effect on RQUICKI levels in Period 2, especially at Week 27, was apparently caused by elevated serum insulin concentration in MYC cows. At Week 27 there was no obvious difference in mean NEFA and glucose levels between groups. Consequently, the higher insulin concentration led to a lower RQUICKI in the MYC group. The higher insulin level in MYC at Week 27 did not lead to decreased lipomobilization and/or enhanced uptake of NEFA in the peripheral tissues according to almost unchanged blood NEFA concentrations, indicating lower peripheral insulin sensitivity in this group compared with control. In ruminants, insulin secretion is stimulated by concentrations of plasma glucose and by volatile fatty acids absorbed from ruminal fermentation [29]. Therefore, the higher mean insulin blood concentration in MYC compared with CON in Period 2 might be related to higher DMI and higher ruminal propionate concentration in these groups as reported by Keese et al. [6] for the cows presented here. However, these mycotoxin effects were found only for the 30% DMI, and for ruminal propionate concentration on the 60% concentrate level. Moreover, no difference was found between the MYC groups and controls in the total ruminal short-chain fatty acid (SCFA) concentrations [6]. Thus, DMI and ruminal SCFA concentrations could explain the higher insulin level in the MYC group only partly.

The BHB blood concentration is influenced by hepatic ketogenesis and production in the ruminal wall, as well as uptake by peripheral tissue. The BHB concentration was generally lower in cows fed on 60% concentrate levels, reflecting lower lipomobilization or reduced hepatic ketogenesis due to higher blood insulin and ruminal propionate concentrations [6]. The reason that blood BHB concentrations in cows of the MYC60 group were lower than MYC30 and CON60 could be due to significantly higher ruminal propionate and lower ruminal butyrate proportions in MYC 60 compared to the other groups [6].

### 3.3. Muscular and Hepatic mRNA Expression of Glucose Transporter 1 (SLC2A1)

The mRNA expression of SLC2A1 (glucose transporter 1), which is seen as an insulin-independent basal glucose transporter [30], was not affected by the dietary intervention in muscular and hepatic tissue. This is in accordance with other studies, where little variation in the expression pattern of SLC2A1 under different dietary conditions, including weaning in young cattle, has been observed [31,32,33].

### 3.4. Muscular mRNA Expression of Glucose Transporter (SLC2A4) and Insulin Receptor (IR)

In muscular tissue, the mRNA transcription of SLC2A4 was downregulated in MYC60 compared with CON60 at Week 27, and that of IR was downregulated in MYC groups compared with control, concomitantly. Moreover, although it was less pronounced, the mRNA transcription pattern of IR was comparable to that of SLC2A4. A lower transcription level of insulin receptors could be associated with a lower density of IR on the cell surface, which may lead to a reduced biological response to insulin, such as cellular glucose uptake through glucose transporter 4, the gene product of SLC2A4. Thus, the observed suppression of IR isoforms and SLC2A4 after 27 weeks of DON exposure may have contributed to reduced insulin sensitivity of peripheral tissue, as assessed by RQUICKI, in our cows. The SLC2A4 transcription, which has been studied intensively, mainly in rodents, differs depending on various factors, including species, organ, as well as tissue components, such as the composition of muscle fibers [34]. Expression of SLC2A4 appears relatively stable against nutritional changes in ruminating cattle [35,36] and in ruminants of the same age and same genetic background against changes in blood insulin or glucose levels [32,37,38]. This is in accordance with our own observation that high dietary concentrate levels solely did not affect SLC2A4 mRNA. However, the results in this study could suggest that an additional metabolic burden, such as chronic intake of mycotoxin contamination in the diet, could lead to the transcriptional modification of SLC2A4 in lactating dairy cows.

### 3.5. Hepatic Insulin Receptor Expression

In liver tissue, we observed a relatively stable transcription of IRA and IRB under nutritional changes. This was also found in a previous study [39], and in a study by Fenwick et al. [40]. Insulin suppresses the hepatic gluconeogenesis and fatty acid oxidation and stimulates the fatty acid esterification in the liver by several mechanisms, including transcriptional regulation. The factors affecting these biological insulin actions are classified at pre-receptor, receptor, and post-receptor levels [22]. In this context, the results indicated that the mycotoxin effect did not occur at the receptor level, at least on the level of mRNA amount. Thus, the mycotoxin-effect found on the hepatic mRNA expression of other investigated genes might be partly explained by the differences of serum insulin concentration (pre-receptor) or those of insulin signal transduction efficiency (post-receptor).

### 3.6. Hepatic mRNA Expression of SLC2A2 and Key Enzymes in Gluconeogenesis and Fatty Acid Metabolism

#### 3.6.1. Lower Expression of Hepatic Enzymes Involved in Gluconeogenesis and Fatty Acid Metabolism in MYC Groups

The mycotoxin-contaminated diet led to a lower hepatic mRNA expression of SLC2A2 (glucose transporter 2) and affected various key enzymes involved in gluconeogenesis (PC, PCK2, G6Pase) and in fatty acid metabolism (CPT1A, ACCA, and GPAM) at Week 27 compared to the control. These results indicated the possibility that a long-term exposure to DON could modify the hepatic energy metabolism at a transcriptional level. However, in contrast to muscle, these mycotoxin effects were not influenced by the dietary concentrate proportion. On the assumption that the level of transcript reflects the functions of investigated enzymes, the observed alteration could have led to a lower hepatic glucose production, a lower fatty acid oxidation, a lower fatty acid synthesis and esterification in mycotoxin-fed cows at Week 27. Nevertheless, we observed that the blood concentrations of glucose, BHB, and NEFA were not lower in MYC groups compared to control groups at Week 27. A possible interpretation could be that the uptake of NEFA, BHB, and glucose in peripheral tissue in MYC groups was reduced at Week 27 compared to control cows.

#### 3.6.2. Possible Explanation for the Suppression of Hepatic SLC2A2 Expression by DON

The gene product of SLC2A2, glucose transporter 2, is a main glucose transporter in the liver [41]. Mutations in SLC2A2 in Fleckvieh cattle [42] caused clinical signs characterized by hepatic glycogen accumulation, which is similar to Fanconi-Bickel syndrome observed in humans with SLC2A2 mutations. These could indicate a comparable clinical impact of SLC2A2 functions between cattle and other species. We observed an increase in the amount of the SLC2A2 transcripts in the liver from Week 0 to 27 in control cows. A similar upregulation was also observed previously, where the hepatic SLC2A2 transcription increased from 21 to 100 days relative to calving, corresponding to Week 0 to 15 in this study [39]. In both studies, the insulin concentration did not alter concomitantly. In mice the SLC2A2 transcription was upregulated by insulin via sterol response element binding protein (SREBP1c) [43]. Thus, these results might indicate that the response of hepatic tissue to the stimulating effect of insulin on the SLC2A2 transcription, possibly via SREBP1c, was enhanced from Week 0 to Week 27 in control cows, however, it did not occur in MYC groups.

#### 3.6.3. Lower Lipogenic Gene Expression in MYC Might Be Due to Reduced Hepatic Insulin Sensitivity

Lower transcription levels of lipogenic genes, ACCA, FASN, GPAM, and DGAT1 at calving and recovery within 21 days after parturition in dairy cows were observed in several studies [44,45,46,47]. Murondoti et al. [48] assumed that this recovery up-regulation of lipogenic genes in early lactation might be related to increasing hepatic NEFA influx and hepatic insulin sensitivity, as well as decreasing leptin blood levels. On this assumption, these genes could have been up-regulated by insulin, possibly via activating the SREBP1c [49]. However, in this study, as discussed above, the hepatic mRNA of ACCA and GPAM were downregulated in MYC groups in Week 27 in spite of higher insulin serum concentration.

#### 3.6.4. Lower mRNA Expression of PC and CPT1A in MYC Might Be Due to Higher Serum Insulin Level

Pyruvate carboxylase (PC) catalyzes the reaction to convert pyruvate to oxaloacetate. The activity of PC correlates positively with its transcription. The transcription of PC is upregulated during periods of inadequate energy supply, because endogenous precursors for gluconeogenesis such as alanine or lactate increase [50]. Studies with lactating cows revealed that transcription of PC is stimulated under reduced insulin/glucagon ratios [51,52,53]. Hammon et al. [54] showed that hepatic PC transcripts correlated negatively with serum insulin concentration in neonatal calves. Thus, the depressed PC transcription in Week 27 in MYC groups appears to be related to their elevated serum insulin concentrations.

Carnitine palmitoyl transferase 1A (CPT1A) catalyzes the transport of cytosolic fatty acids into mitochondria, the rate-limiting step in fatty acid oxidation [46,55]. The transcription of CPT1A is downregulated by insulin via decreasing cAMP levels, while inhibitory effects on activity of CPT1A occur via malonyl-CoA synthesis through stimulating the acetyl-CoA carboxylase [55]. Therefore, again, lower mRNA expression of CPT1A in MYC groups was possibly due to the higher levels of serum insulin in these cows compared to control cows.

#### 3.6.5. Changes in Hepatic Insulin Sensitivity May Be Related to Hepatic Gene Expression

Regarding the hepatic insulin sensitivity, a previous study showed that the suppressive effects of insulin on hepatic glucose production appeared to be present at 100 days after parturition (d100), but not at d21 [39]. It was concluded that the hepatic responses to insulin, assessed by insulin-induced phosphorylation of forkhead box O1 (FoxO1)—a key transcription factor for G6Pase and PCK1—increased from early- to mid-lactation [39,56]. On this assumption, the increase in the hepatic SLC2A2 transcript in Week 27 in control groups in this study could indicate the increase in the hepatic response to insulin from Week 15 to Week 27. This could also explain the results found in lipogenic genes, ACCA and GPAM, that presented concomitant upregulation in control groups, because they are upregulated by insulin through SREBP1c [49,57]. Therefore, the results could be interpreted that in mycotoxin-fed groups, this physiological upregulation of hepatic SLC2A2, ACCA, and GPAM, along with the increase in insulin sensitivity after parturition, could be diminished by long-time exposure to DON. On the other hand, regarding the genes involved in gluconeogenesis and fatty acid oxidation, it was likely that the observed lower expression of affected genes (PC and CPT1A) at Week 27 in MYC groups was due to the suppressive effect of higher levels of insulin in this group. In short, the suppressing effects of insulin on gluconeogenesis and ketogenesis appeared to not be affected by DON, while the stimulating effect of insulin on fatty acid synthesis and esterification might be disturbed by the mycotoxin-contaminated diet. The reason for this controversy could not be elucidated in this study. It might be explained by the complexity of metabolic regulation, including the insulin signaling pathway. It might be possible that DON suppressed only the pathway of insulin-signaling specific for fatty acid synthesis and esterification, for example, via SREBP1c through atypical protein kinase C (aPKC), while the other pathway, which leads to the suppression of gluconeogenesis and fatty acid oxidation through inactivation, e.g., through protein kinase B (Akt) and FoxO1, was not affected. Pathway-specific disturbance of insulin signaling was observed in hepatic insulin resistance in human hepatic steatosis [57].

### 3.7. Possible Mechanisms of DON Effects on Insulin Sensitivity

In cows, about 16% of ingested DON reaches the small intestine with less than 1% systemic circulation due to ruminal degradation of DON to DOM-1 [58]. Accordingly, very low mean serum levels of DON were found in the cows fed a diet contaminated on a level of about 4.5 mg/kg DM with DON (dosage: about 0.15 mg DON/kg BW). Mean DON serum concentrations were, on average, 2–4 ng/mL (range 0.0–18.0 ng/mL) in Period 1 and, in Period 2, were even lower and, in the majority of cows, close to the detection limit [59]. Repeated exposure to low DON doses may have induced inflammatory reactions with activation of MAPK, which may result in enhanced expression of proinflammatory cytokines and tumor necrosis factor alpha (TNFa) in male B6C3F1 mice [60], reviewed in [2] and subsequently in reduced insulin sensitivity [61]. However, such cumulative effects of DON would most likely occur within days after exposure and can, therefore, only partly explain the changes in insulin sensitivity observed after long-term exposure of 27 weeks in the MYC cows. Unfortunately, cytokines have not been measured in this study. Keese et al. [6] found changes in the ruminal fermentation pattern of cows fed a DON-contaminated diet. Compared to controls, reduced molar percentages of ruminal acetate and butyrate concentrations were found, whereas the molar percentage of propionate increased. These observations were unlikely attributable only to differences in DMI, but indicate probable changes in the ruminal microbiome possibly due to direct antimicrobial mycotoxin effects and/or indirect effects of the *Fusarium* infection-related alteration in the physio-chemical properties of the infected cereal on ruminal microbes. However, the effects of changes in the ruminal microbiome on energy metabolism needs further evaluation in dairy cows [62]. DON treatment can cause oxidative stress with the generation of reactive oxygen and nitrogen species [63]. Recently, it was shown that oxidative stress may contribute to the development of insulin resistance in cows [64]. Furthermore, experiments in humans and experimental animals have also shown that DON may disrupt gut integrity, inhibit intestinal absorption of nutrients, and affect the secretion of gut hormones and the growth hormone axis [15,16]. However, appropriate studies in cows on this are still missing. In summary, a conclusive explanation for the effects on insulin sensitivity after a long-term exposure of 27 weeks to a DON-contaminated diet in cows cannot be derived from the results of this study.

## 4. Conclusions

The results of this study revealed mild effects of a long-term dietary DON exposure (27 weeks) at a dose of about 5 mg DON/kg dry matter (approximately 0.2 mg/kg body weight per day) on energy metabolism in dairy cows. Reduced mRNA expression of muscular SLC2A4 and IR may reflect reduced insulin sensitivity in DON exposed cows on a molecular level. Long-lasting dietary DON exposure also appears to affect the transcription of hepatic gluconeogenetic enzymes and may affect hepatic fatty acid metabolism by insulin-stimulated downregulation of PC and CPT1A, whereas fatty acid synthesis (ACCA) and esterification (GPAM) was also downregulated by dietary DON intake in spite of higher serum insulin levels. The study revealed no consistent evidence that DON effects were modified by high dietary concentrate levels.

## 5. Materials and Methods

The experiments were conducted according to the European Community regulations concerning the protection of experimental animals and the guidelines of the LAVES (Lower Saxony State Office for Consumer Protection and Food Safety, Germany, file number 33.42502-4/09-01.03, approval date 28 March 2003). The feeding study was performed at the Institute of Animal Nutrition, Friedrich-Loeffler-Institute (FLI) in Braunschweig, Germany. The effects on performance parameters, ruminal fermentation, and parameters of the acid-base metabolism were published elsewhere [6,23,59].

### 5.1. Animals and Feeding

The general experimental design has been described in detail elsewhere [23]. In brief, 27 German Holstein cows (BW 522 ± 56 kg, 31 days in milk (DIM) on average, 12 pluriparous, and 15 primiparous) were used. During Period 1 (0–11 experimental weeks) the animals were randomly assigned to two groups; A control group (CON) with 14 animals and a mycotoxin-fed group (MYC) with 13 animals. Diets contained 50% concentrate and 50% roughage on a dry matter basis (Table 6). The mycotoxin-contaminated diet offered to MYC included 5.3 mg DON kg DM and 113 µg ZEN/kg DM on average. The average daily DON intake by cows was 18 µg/kg and 187 µg/kg BW in the CON and MYC group, respectively [23]. During Period 2 (12–27 experimental weeks) the original MYC and CON groups were further divided in two subgroups and fed different concentrate levels in the ration. Cows in the CON group in Period 1 were randomly divided into CON30 and CON60 (30% and 60% concentrates, *n* = 7 for each), cows in the MYC group in Period 1 were randomly divided into MYC30 (30% concentrates, 4.4 mg DON/kg DM, *n* = 6) and MYC60 (60% concentrates, 4.6 mg DON/kg DM, *n* = 7). At the beginning of Period 2, the mean BW was 565 ± 33 kg in CON30, 574 ± 42 kg in MYC30, 593 ± 100 kg in CON60, and 569 ± 49 kg in MYC60. The mean DIM was 99. The diet components and mycotoxin contents are presented in Table 6. The average daily DON intake by cows was 17.0, 13.6, 123, and 154 µg/kg BW in the CON30, CON60, MYC30, and MYC60, respectively [23].

The animals were housed in a free-stall with unrestricted access to diet and water, being separated according to feeding groups. Diets were offered as a partial mixed ration ad libitum every morning at 10:30 a.m. after milking. Concentrate was offered separately at automatic feeding stations. Rations were isoenergetic, and the concentration of mycotoxins in the rations for MYC group in Period 1 and Period 2 were similar.

### 5.2. Collection of Blood and Tissue Samples

Blood samples were taken on experimental weeks 0 (baseline), 4, 8, 15, 19, 21, and 27 from the jugular vein into native tubes and tubes coated with Li-heparin and Na-fluoride. Samples were centrifuged for 15 min at 5 °C, 1500× *g*, and serum and plasma aliquots were stored at −80 °C until further analysis. Hepatic and muscular biopsies were taken from five cows of each group under aseptic conditions on experimental weeks 0, 15, and 27. Biopsy sites were shaved, washed, degreased with medical alcohol, and disinfected with iodine (Betaisodona^®^ 10%, Albrecht GmbH, Aulendorf, Germany).

After caudal epidural anesthesia with 5 mL procaine (WDT, Garbsen, Germany) and infiltration of the incision line with 5 mL procaine muscle biopsies were taken in the region 8 cm below the ischiadic tuber from the caudal fascial compartment of the M. semitendinosus, M. biceps femoris, or M. semimembranosus. The sampling was performed alternating on the left or right thigh. Through a 2 cm incision about 1500 mg of muscular tissue were taken. Skin lesions were closed using a commercial suture material (Supramid^®^, Albrecht GmbH, Aulendorf, Germany) and locally treated with 2.5 mL Procain-Penicillin (Albrecht GmbH, Aulendorf, Germany).

After infiltration anesthesia of the abdominal wall with 5 mL procaine liver biopsies were obtained from the 9th or 10th intercostal space transcutaneously under ultrasonographic control with an automatic device for biopsy sampling (Biopsy, C. R. Bard, Inc., Tempe, AZ, USA) and commercial Tru-Cut biopsy needles (Albrecht GmbH, Aulendorf, Germany).

Tissue samples were rinsed with saline to remove blood contamination, shock-frozen in liquid nitrogen, and stored at −80 °C until analysis.

### 5.3. Analyses of Blood Samples

Concentration of glucose (MTI-Diagnostics GmbH, Idstein, Germany) in fluoride plasma and non-esterified fatty acid (NEFA) (WAKO Chemicals GmbH, Neuss, Germany) and β-hydroxybutyrate (BHB) (Randox Laboratories GmbH, Krefeld, Germany) in serum were measured spectrophotometrically using an automatic clinical chemistry analyzer (Cobas Mira Plus, Roche, Basel, Switzerland) and commercial kits. Serum insulin concentrations were measured by means of radioimmunoassay using a commercial test kit (The DSL-1600 INSULIN RIA Kit, Diagnostic Systems Laboratories, Inc., Webster, TX, USA). The detection limit of the insulin assay was 1.3 µU/mL. The revised quantitative insulin sensitivity check index (RQUICKI) was calculated according to Holtenius and Holtenius [19] (RQUICKI = 1/[log(glucose in mg/dL) + log(insulin in μU/mL) + log(NEFA in mmol/L)]).

### 5.4. Analyses of Tissue Samples

#### 5.4.1. RNA Isolation

About 50–80 mg of muscle and about 25–50 mg of liver tissue was homogenized in 500 µL Trizol^®^ (Invitrogen, Carlsbad, CA, USA) with a FastPrep Homogenizer and Isolation System (Thermo Scientific, Waltham, MA, USA) at the speed of 6.0 m/sec for maximum 2 × 30 s. After 5 min incubation at room temperature (RT) 100 µL chloroform was added. Samples were vortexed for 15 s, and incubated at RT for 10 min followed by centrifugation (Biofuge^®^ Fresco, Heraeus, Hanau, Germany) at 4 °C and 12,000× *g* for 15 min. The upper aqueous part containing the RNA was transferred to a new 1.5 mL tube. RNA was precipitated by adding 0.25 mL of 100% isopropyl alcohol (4 °C). The tubes were inverted ten times by hand and incubated at RT for 10 min. Samples were centrifuged at 4 °C and 9200× *g* for 8 min. After being dried at RT for 10 min, pellets were dissolved in water (Ampuwa, Fresenius Kabi, Bad Homburg vor der Höhe, Germany; 30 μL for muscle and 60 μL for liver).

Concentration of the isolated RNA was estimated by measuring the absorbance at a wavelength of 260 nm using a biospectrophotometer (Eppendorf, Hamburg, Germany). The ratio A260/280 was calculated to check for protein contamination. The integrity of the RNA was examined by lab-on-chip capillary gel-electrophoresis according to the manufacturer’s instruction (Agilent 2100 Bioanalyzer, Agilent RNA 6000 Nano Assay, Agilent Technologies, Santa Clara, CA, USA). Only the RNA samples with the RNA integrity number more than 6.0 and A260/280 ratio more than 1.9 were used for further analysis.

#### 5.4.2. Reverse Transcription

The samples including 1.5 µg RNA were mixed with 1.5 µL of DNase (RQ1 RNase-Free DNase, Promega, Madison, WI, USA) and water to a final volume of 10 µL, incubated at 37 °C for 30 min for the digestion of genomic DNA, followed by an incubation at 75 °C for 10 min for the inactivation of the enzyme. Samples containing 1 µg of DNase-treated RNA were added to the reaction-mix containing 2.5 unit MULV-reverse transcriptase (Applied Biosystems, Waltham, MA, USA), one unit of RNase inhibitor (Applied Biosystems, Waltham, MA, USA), 1 mM dNTPs (BioRad Laboratories, Inc., Hercules, CA, USA), 2.5 µM random hexamers (Applied Biosystems, Waltham, MA, USA), 5 mM MgCl_2_ (Invitrogen, Carlsbad, CA, USA), PCR buffer with 50 mM KCl and 10 mM Tris-HCl (pH 8.3; Applied Biosystems, Waltham, MA, USA), and water. The mixture was incubated at 25 °C for 10 min., at 42 °C for 60 min. and at 99 °C for 5 min on a thermocycler (TProfessional Standard 96, Biometra GmbH, Göttingen, Germany). The synthesized complementary DNA (cDNA) was diluted with water at 1:1 or 1:3 and stored at −20 °C until analysis.

#### 5.4.3. Real-Time qPCR Assay

Real-time quantitative polymerase chain reaction (real-time qPCR) was conducted using SYBR Green Supermix (Biorad Laboratories, Inc., Hercules, CA, USA) and the iCycler iQ5 Real Time PCR Detection System (Biorad, Laboratories, Inc., Hercules, CA, USA). The final reaction volume was 20 µL. Each reaction contained 0.5–1 µL of cDNA template from the original reverse transcript reaction and 0.5–1 µmol/L primers. Cycling parameters were 95 °C for 3 min., 40 cycles of 95 °C for 30 s, 60 °C for 30 s, and at 72 °C for 30 s with signal acquisition, followed by a melting curve analyses within the range from 55–95 °C. The assay was performed in triplicate. Each assay contained non-template control and standards for the standard curve and inter-run calibrator.

The investigated genes in the muscle samples were glucose transporter 1 (SLC2A1), glucose transporter 4 (SLC2A4), and insulin receptor isoform A and B (IRA and IRB). The investigated genes in the liver samples were SLC2A1, glucose transporter 2 (SLC2A2), IRA, IRB, key enzymes for gluconeogenesis–fructose-1,6-bisphosphatase 1 (FBP1), glucose-6-phosphatase (G6Pase), phosphoenolpyruvate carboxykinase 1 and 2 (PCK1 and PCK2), pyruvate carboxylase (PC), propionyl-CoA carboxylase, alpha polypeptide (PCCA)–, key enzymes for ketogenesis–carnitine palmitoyltransferase 1A (CPT1A), acyl-CoA synthetase long-chain family member 1 (ACSL1), 3-hydroxy-3-methylglutaryl-CoA synthase 2 (HMGS2), 3-hydroxymethyl-3-methylglutaryl-CoA lyase (HMGL), 3-hydroxybutyrate dehydrogenase, type 2 (BDH2)–, key enzymes for fatty acid synthesis and esterification–acetyl-CoA carboxylase (ACCA), fatty acid synthase (FASN), mitochondrial glycerol phosphate acyltransferase (GPAM), and diacylglycerol O-acyltransferase homolog 1 (DGAT1). Characteristic of primers and accession numbers in GenBank for the investigated genes of interest are listed in Table 7. All primers were synthesized by Eurofins Genomics GmbH (Ebersberg, Germany). Primer sequences for primers for IRA and IRB [65] and mitochondrial ribosomal protein S15 (MRPS15) and ubiquitously-expressed transcript (UXT) [66] were selected according to the literature. The information about the primers and assays for SLC2A2, IRA, IRB, G6Pase, PCCA, PYGL, RPS9, MRPS15, and UXT were already published [39]. Primer sequences for the other genes were designed using Primer3 (http://primer3.sourceforge.net/webif.php) [67]. Each primer pair was selected according to the following criteria: (1) targeted sequence includes an exon/intron boundary; (2) the length of the PCR-products is 150–250 bases; (3) the melting temperature of the primer is 60 °C; and (4) the guanine-cytosine content of each primer sequence is 50%. Single amplification products and no primer dimer formation were confirmed with single melting temperatures, as well as single discrete bands on the 2% agarose gel electrophoresis. The specificity of the PCR-products was further tested by sequencing (Eurofins Genomics GmbH, Ebersberg, Germany) followed by BLAST analysis [68]. All the sequences of PCR products were found specifically in the target sequences (Table S1).

Reference genes were selected by analyzing the stability of expression of four candidate genes—UXT, ribosomal protein S9 (RPS9), MRPS15, and β-glucuronidase (GUSB)—in 16 samples taken from every group and time. The geNorm analysis performed using qBase Plus (Biogazelle NV, Zwijnaarde, Belgium) revealed that MRPS15 and UXT (average expression stability value M = 0.4254) were expressed most stably across the muscle samples, and RPS9 and UXT (M = 0.244) in liver samples. The further results of the qPCR assay were analyzed also using qBase Plus. Normalized relative quantities of RNA for target genes were calculated from the threshold cycles (Cq) at relative fluorescence units of 50 using the delta-delta-Cq method modified for the use of multiple reference genes for normalization [69]. Amplification efficiency was calculated from standard curves, made from four step dilution series of pooled cDNA of all samples.

### 5.5. Statistical Analysis

Data was analyzed using SAS (SAS institute Inc., Version 9.1, Cary, NC, USA) statistics software. The effect of experimental week, mycotoxin, concentrate levels, and their interactions were evaluated using the MIXED procedure with repeated measures [70] and the restricted maximum likelihood method. The mixed models were fitted in each period for all the analyzed blood variables and RQUICKI. The variables from the analysis of biopsy samples were analyzed for the whole experimental period, because only one sample in Week 0 was taken in Period 1. The fixed factors included were M (mycotoxin), W (experimental week), and their interactions for Period 1 and M, C (concentrate levels), W, and their interactions for Period 2. Cows within experimental groups were used as a subject in the REPEATED statement. The considered covariance structures were composed symmetric, variance component, ante-dependence, and unstructured. Model optimization was performed using Akaike’s information criterion considering covariance structures and random effects. Kenward-Roger adjustment was applied to calculate the degrees of freedom. In the initial model, cows within experimental groups and lactation number (pluri- and primiparous) were considered to constitute random effects. They were removed during model optimization when needed. The appropriateness of the mixed model assumptions was examined using residuals analysis. For BHB, NEFA, and insulin the statistical evaluation was performed on logarithmized values for the normality of the residual distribution. Least square means (LSM) were calculated separately on the factor M × W for Period 1 and on the factor M × C × W for Period 2. Multiple comparisons of LSM were performed by PDiff. The calculated *p*-values were adjusted according to Holm’s method [71] within the variable and a period using the R [72] “p.adjust” function in the package “stats”. All tests were two-tailed. Significances and trends for differences of LSM were decided under adjusted probabilities <0.05 and <0.1, respectively.

## Figures and Tables

**Table 1:** Blood metabolites and RQUICKI as affected by mycotoxin-contaminated diet (overall group means and results of analysis of variances for each period).

	Period 1						Period 2										
	Group (*n*) ^2^			*p*-Values of Fixed Effects			Group (*n*) ^2^					*p*-Values of Fixed Effects					
	CON (14)	MYC (13)	SEM	W	M	W × M	CON30 (7)	CON60 (7)	MYC30 (6)	MYC60 (7)	SEM	W	M	W × M	C	W × C	M × C × W
**Glucose** (mmol/L)	3.53	3.43	0.06	0.013	0.19	0.35	3.95	3.96	3.69	3.89	0.10	<0.001	0.12	0.58	0.28	0.79	0.35
**NEFA ^1^** (log10, µmol/L)	2.26	2.25	0.02	0.002	0.80	0.87	2.33 ^a^	2.11 ^b^	2.21 ^c^	2.14 ^b,c^	0.03	0.23	0.21	0.27	<0.001	0.035	0.30
**BHB ^1^** (log10, µmol/L)	2.70	2.72	0.03	0.061	0.60	0.96	2.54	2.50	2.67	2.49	0.05	<0.001	0.23	0.019	0.031	0.40	0.001
**Insulin ^1^** (log10, µU/mL)	0.57	0.59	0.03	<0.001	0.66	0.91	0.43	0.72	0.67	0.81	0.06	0.017	0.016	0.45	0.002	0.24	0.71
**RQUICKI**	0.65	0.64	0.02	0.005	0.62	0.55	0.66	0.61	0.59	0.57	0.02	<0.001	0.048	0.60	0.17	0.97	0.36

^1^ The statistical analysis was performed after logarithmic transformation of raw values; ^2^ Group means throughout the Period 1 and 2; SEM: pooled standard error of means; BHB: beta-hydroxybutyrate; NEFA: non-esterified fatty acid; RQUICKI: revised quantitative insulin sensitivity check index; W: time effect (experimental week effect); M: mycotoxin effect; C: effect of concentrate proportion in the diet; W × M, W × C, M × C × W: interaction effects of W, M, and C; CON: control group; MYC: mycotoxin group; CON30 or CON60: control group with 30% or 60% concentrate proportion in the diet; MYC30 or MYC60: mycotoxin group with 30% or 60% concentrate proportion in the diet. “abc”: Corresponding group means with different superscript letters were significantly different within the week (adjusted *p* < 0.05).

**Table 2:** Muscular mRNA expression of glucose transporter 1 and 4 (SLC2A1 and 4) and insulin receptors (IRA and B) (group means in each sampling week and the results of analysis of variances).

Gene	Sampling Week	Group (*n* = 5/Group) ^1^					*p*-Values of Fixed Effects ^2^					
		CON30	CON60	MYC30	MYC60	SEM	W	M	W × M	C	W × C	W × C × M
SLC2A1	0	5.98	10.24	7.70	9.08	3.26	<0.001	0.013	0.16	0.72	0.64	0.21
	15	4.37	6.87	18.69	9.32	2.09						
	27	2.55	4.19	5.43	4.05	1.11						
SLC2A4	0	4.67	3.67	5.29	4.38	0.92	<0.001	0.36	0.30	0.24	0.55	0.007
	15	8.95	6.69	6.69	6.72	0.92						
	27	3.82 ^ab^	6.99 ^b^	5.74 ^ab^	2.93 ^a^	0.92						
IRA	0	4.36	4.26	4.36	3.86	0.71	0.16	0.32	0.002	0.10	0.11	0.081
	15	3.81	4.29	5.10	5.65	0.69						
	27	3.04	6.88	2.66	2.46	0.69						
IRB	0	4.55	4.88	5.11	4.15	0.71	<0.001	0.039	0.001	0.32	0.11	0.090
	15	4.74	5.95	6.61	5.20	0.69						
	27	3.06	7.39	2.62	2.21	0.69						

^1^ CON30 or CON60: control group with 30 or 60% concentrate proportion in the diet; MYC30 or MYC60: mycotoxin group with 30 or 60% concentrate proportion in the diet. ^2^ W: time effect (experimental week effect); M: mycotoxin effect; C: effect of concentrate proportion in the diet; W × M, W × C, M × C × W: interaction effects of W, M, and C; “ab”: Corresponding group means with different superscript letters were significantly different within the week (adjusted *p* < 0.05). SEM: standard error of mean.

**Table 3:** Hepatic mRNA expression of glucose transporter 1 and 2 (SLC2A1 and 2) and insulin receptor A and B (IRA and B) (group means in each sampling week and the results of analysis of variances).

Gene	Sampling Week	Group (*n* = 5/Group) ^1^					*p*-Values of Fixed Effects ^2^					
		CON30	CON60	MYC30	MYC60	SEM	W	M	W × M	C	W × C	W × C × M
SLC2A1	0	3.39	3.29	4.67	4.72	0.58	0.25	<0.001	0.83	0.43	0.47	0.80
	15	3.56	4.41	4.82	5.7	0.58						
	27	2.95	3.35	5.12	4.67	0.58						
SLC2A2	0	4.25	5.47	3.95	5.61	1.32	0.005	0.038	0.009	0.58	0.39	0.52
	15	7.16	8.03	6.63	7.70	1.32						
	27	11.83	8.99	4.56	5.54	1.32						
IRA	0	4.47	4.78	3.88	3.82	0.62	0.15	0.55	0.21	0.22	0.40	0.73
	15	4.25	2.69	4.44	3.87	0.62						
	27	3.86	3.43	3.28	2.91	0.62						
IRB	0	2.19	3.08	2.65	2.96	0.35	0.52	0.58	0.56	0.68	0.20	0.34
	15	3.17	2.73	2.43	2.72	0.35						
	27	3.06	3.05	3.18	2.66	0.35						

^1^ CON30 or CON60: control group with 30% or 60% concentrate proportion in the diet; MYC30 or MYC60: mycotoxin group with 30% or 60% concentrate proportion in the diet. ^2^ W: time effect (experimental week effect); M: mycotoxin effect; C: effect of concentrate proportion in the diet; W × M, W × C, M × C × W: interaction effects of W, M, and C; SEM: standard error of mean.

**Table 4:** Hepatic mRNA expression of key enzymes for gluconeogenesis (group means in each sampling week and the results of analysis of variances).

Gene	Sampling Week	Group (*n* = 5/Group) ^1^					*p*-Values of Fixed Effects ^2^					
		CON30	CON60	MYC30	MYC60	SEM	W	M	W × M	C	W × C	W × C × M
G6Pase	0	6.68	6.48	5.55	7.13	1.14	<0.001	0.60	0.051	0.41	0.023	0.89
	15	6.94	5.66	8.45	10.17	1.14						
	27	11.91	8.65	9.59	7.62	1.14						
FBP1	0	5.67	6.65	4.26	5.83	1.46	<0.001	0.027	0.13	0.53	0.23	0.40
	15	9.07	6.49	6.69	8.20	1.46						
	27	15.27	9.83	7.79	8.56	1.46						
PC	0	24.21	32.37	35.87	32.11	0.67	<0.001	0.35	0.021	0.61	0.55	0.35
	15	19.65	14.51	15.82	12.10	3.62						
	27	21.98	14.70	5.13	5.88	3.03						
PCK1	0	12.80	14.75	14.31	17.11	2.35	<0.001	0.69	0.61	0.073	0.012	0.84
	15	18.80	10.37	16.71	10.40	2.35						
	27	6.86	4.06	8.53	4.23	2.35						
PCK2	0	3.42	3.94	4.78	4.77	0.59	0.005	0.69	0.017	0.12	0.25	0.84
	15	4.98	4.04	5.95	5.31	0.59						
	27	6.62	5.81	5.85	4.44	0.59						
PCCA	0	2.57	3.42	2.73	3.43	0.67	<0.001	0.69	0.28	0.46	0.093	0.43
	15	4.42	3.63	4.90	4.96	0.67						
	27	6.07	5.42	6.32	4.23	0.67						
PYGL	0	6.77	6.84	5.70	8.54	1.25	0.20	0.88	0.70	0.50	0.37	0.35
	15	16.26	5.25	9.83	12.92	4.37							
	27	11.67	5.57	5.35	8.21	2.28							

^1^ CON30 or CON60: control group with 30 or 60% concentrate proportion in the diet; MYC30 or MYC60: mycotoxin group with 30 or 60% concentrate proportion in the diet. ^2^ W: time effect (experimental week effect); M: mycotoxin effect; C: effect of concentrate proportion in the diet; W × M, W × C, M × C × W: interaction effects of W, M, and C; G6Pase: glucose-6-phosphatase; FBP1: fructose-1,6-bisphosphatase 1; PCK1 and PCK2: phosphoenolpyruvate carboxykinase 1 and 2; PC: pyruvate carboxylase; PCCA: propionyl-CoA carboxylase, alpha polypeptide; PYGL: glycogen phosphorylase, liver; SEM: standard error of mean.

**Table 5:** Hepatic mRNA expression of key enzymes for fatty acid oxidation and esterification (group means in each sampling week and the results of analysis of variances).

Gene	Sampling Week	Group (*n* = 5/Group) ^1^					*p*-Values of Fixed Effects ^2^					
		CON30	CON60	MYC30	MYC60	SEM	W	M	W × M	C	W × C	W × C × M
ACSL1	0	2.41	3.57	1.96	2.10	0.51	0.83	0.049	0.18	0.70	0.45	0.15
	15	3.04	2.20	2.58	3.02	0.51						
	27	3.67	2.74	1.82	2.55	0.51						
CPT1A	0	4.86	5.67	4.82	6.09	0.76	0.096	0.37	0.032	0.83	0.031	0.60
	15	4.37	4.01	4.43	5.27	0.72						
	27	7.86	4.98	3.89	3.25	1.26						
HMGS2	0	5.09	6.12	5.30	7.10	1.39	<0.001	0.13	0.56	0.44	0.099	0.52
	15	8.60	6.45	10.21	8.00	1.39						
	27	10.11	10.06	14.11	11.02	1.39						
HMGL	0	26.01	28.92	24.11	30.73	0.50	<0.001	0.41	0.071	0.11	0.074	0.38
	15	44.76	25.48	45.21	48.37	0.50						
	27	51.03	36.67	42.93	35.95	0.50						
BDH2	0	3.11	2.78	4.70	3.18	0.75	<0.001	0.50	0.35	0.013	0.15	0.50
	15	4.74	3.31	5.18	3.94	0.75						
	27	7.23	4.14	6.44	4.30	0.75						
ACCA	0	3.89	5.05	5.79	3.78	1.63	0.001	0.35	<0.001	0.24	0.91	0.091
	15	9.42	4.28	9.53	9.68	1.63						
	27	12.53	11.56	6.45	5.07	1.63						
FASN	0	3.72	2.09	4.44	2.43	0.77	0.002	0.74	0.19	0.49	0.40	0.65
	15	6.65	5.42	6.64	8.19	1.79						
	27	6.83	6.09	3.50	3.77	1.61						
GPAM	0	5.92	7.81	5.43	6.74	1.14	0.026	0.51	0.002	0.047	0.91	0.26
	15	7.40	6.06	7.94	11.18	1.14						
	27	9.72	10.92	6.05	7.87	1.14						
DGAT1	0	1.48	1.90	2.12	1.67	0.15	0.001	0.64	0.14	0.36	0.48	0.20
	15	2.09	1.91	2.42	2.19	0.30						
	27	2.20	2.23	2.19	1.68	0.16						

^1^ CON30 or CON60: control group with 30 or 60% concentrate proportion in the diet; MYC30 or MYC60: mycotoxin group with 30 or 60% concentrate proportion in the diet. ^2^ W: time effect (experimental week effect); M: mycotoxin effect; C: effect of concentrate proportion in the diet; W × M, W × C, M × C × W: interaction effects of W, M, and C. ACSL1: acyl-CoA synthetase long-chain family member 1; CPT1A: carnitine palmitoyltransferase 1A; HMGS2: 3-hydroxy-3-methylglutaryl-CoA synthase 2; HMGL: 3-hydroxymethyl-3-methylglutaryl-CoA lyase; BDH2: 3-hydroxybutyrate dehydrogenase, type 2; ACCA: acetyl-CoA carboxylase; FASN: fatty acid synthase; GPAM: mitochondrial glycerol phosphate acyltransferase; DGAT1: diacylglycerol O-acyltransferase homolog 1; SEM: standard error of mean.

**Table 6:** Ingredients and nutrient components of diets ^1^.

	Period 1		Period 2			
	CON	MYC	CON30	CON60	MYC30	MYC60
Ingredients (%)						
Triticale	25	10.5	15	30	6.3	12.6
Fusarium-contaminated triticale	0	14.5	0	0	8.7	17.4
Soybean meal	13.35	13.35	8.01	16.02	8.01	16.02
Maize	10.35	10.35	6.21	12.42	6.21	12.42
Mineral feed	0.7	0.7	0.42	0.84	0.42	0.84
Calcium carbonate	0.6	0.6	0.36	0.72	0.36	0.72
Maize silage	25	25	35	20	35	20
Grass silage	25	25	35	20	35	20
Dry matter [g/kg]	452	465	386	456	394	475
Nutrients [g/kg DM]						
Crude ash	6	61	68	61	69	66
Crude protein	150	153	135	165	139	154
Crude fat	25	26	28	26	28	27
Crude fiber	143	143	203	142	196	128
Acid detergent fiber	157	157	220	161	212	169
Neutral detergent fiber	305	307	391	349	394	349
Metabolizable energy [MJ/kg DM]	11.6	11.6	11.4	12.4	11.6	12.1
Net energy lactation [MJ/kg DM]	7.1	7.1	7.0	7.8	7.1	7.5
Mycotoxins						
Deoxynivalenol [mg/kg DM]	0.6	5.3	0.6	0.4	4.4	4.6
Zearalenone [µg/kg DM]	63.1	112.7	35.0	24.4	73.8	72.5

^1^ According to [6]. DM: dry matter, CON: control group, MYC: mycotoxin group, CON30 or CON60: control group with 30% or 60% concentrate proportion in the diet, MYC30 or MYC60: mycotoxin group with 30% or 60% concentrate proportion in the diet.

**Table 7:** Characteristics of the primers and the real-time qPCR conditions.

	Primer Sequences (5′-3′) ^2^	Accession Number	Size (Base) ^3^	Tm (°C) ^4^	Tissue ^5^	Linear Dynamic Range(ng RNA) ^6^	E ^7^
SLC2A1 ^1^	F-AGATGATGCGGGAGAAGAAG	BC119940	238	89	L	6.25–100	1.86 (0.98)
	R-TCCACCACAAATAGCGACAC				M	6.25–100	1.99 (0.95)
SLC2A4	F-CCAACTGGACATGCAACTTCATC	BC114082	206	85	M	3.13–67.5	1.90 (0.99)
	R-CACTTCCTGCTCCAGAAGAGA						
FBP1	F-CAACATCGACTGCCTTGTGT	BC102974	224	88	L	1.56–50	1.97 (0.99)
	R-CCCTGTCCACCAAAATGAAC						
PCK1	F-AACTCGCTCCTTGGGAAGAA	BC112664	261	89	L	3.13–50	1.91 (0.99)
	R-CCGCAAGTTACCTTGTTGGT						
PCK2	F-CATTGACGCCATCATCTTTG	BC102244	150	88	L	3.13–50	1.87 (0.99)
	R-TGCATGATGACCTTCCCTTT						
PC	F-CTATGTTGCCCACAGCTTCA	BC114135	244	90	L	3.13–50	1.93 (0.98)
	R-GATGTCCATGCCATTCTCCT						
CPT1A	F-CCTATTTTGGACACGGGAAA	XM_002699420.1	172	86.5	L	3.13–50	1.78 (0.99)
	R-TCAAACCACCTGTCGAAACA						
HMGS2	F-GGCCTTTCACTCTCGATGAT	BC112666	170	85	L	1.56–50	1.93 (0.99)
	R-TTCCAGCTTTAGTCCCCTGA						
HMGL	F-CAGCTTCGTGTCTCCCAAAT	BC118276	227	87	L	3.13–50	1.87 (0.99)
	R-ACCGCTGCAAACTCTCATCT						
BDH2	F-TATCCGGTGCAACTGTGTGT	BC102567	204	84	L	3.13–50	2.01 (0.98)
	R-ACAGGATTCCCCGTGATGTA						
ACCA	F-ATCCGACGCCTTACTTTCCT	AJ132890	193	82.5	L	3.13–50	2.05 (0.98)
	R-TTCTCATCCGGTTCAGCTCT						
FASN	F-AACACCTCGGTGCAGTTCAT	AY343889	196	88	L	3.13–50	1.94 (0.99)
	R-CTTTCTCTTTGCAGCCATCC						
GPAM	F-GCCAGATGGACGGAAAGATA	AY515690	182	83.5	L	3.13–50	2.00 (0.97)
	R-TCGTATTGGTGGACAGCGTA						
DGAT1	F-CTCTGTGCCTGGTCATTGTG	BC118146	239	90	L	3.13–50	1.89 (0.99)
	R-CCGGTAGGAGAACAGCTTGA						
ACSL1	F-GAACTACAGGCAACCCCAAA	BC119914	240	83	L	3.13–50	1.94 (0.99)
	R-GGGCCTTGAGATCATCCATA						
GUSB	F-ACCAGAAGCCGATGATTCAG	BC133415	160	84	L	3.13–75	1.96 (0.99)
	R-AGCTCGCCAACCACATATTC				M	6.25–100	1.76 (0.96)

SLC2A1: glucose transporter 1, FBP1: fructose-1,6-bisphosphatase 1, PCK1 and PCK2: phosphoenolpyruvate carboxykinase 1 and 2, PC: pyruvate carboxylase, CPT1A: carnitine palmitoyltransferase 1A, ACSL1: acyl-CoA synthetase long-chain family member 1, HMGS2: 3-hydroxy-3-methylglutaryl-CoA synthase 2, HMGL: 3-hydroxymethyl-3-methylglutaryl-CoA lyase, BDH2: 3-hydroxybutyrate dehydrogenase, type 2, ACCA: acetyl-CoA carboxylase, FASN: fatty acid synthase, GPAM: mitochondrial glycerol phosphate acyltransferase, DGAT1: diacylglycerol O-acyltransferase homolog 1, GUSB: β-glucuronidase. ^1^ Primer concentration and annealing temperature for this gene was 1.0 µM and 58 °C; ^2^ F-forward, R-reverse, Exon-exon junctions are underlined; ^3^ Amplicon sizes of PCR products; ^4^ Melting temperatures of PCR products; ^5^ Tissues which were measured for the gene. L: liver, M: muscle; ^6^ The range of RNA input (ng) in which linearity of standard curve was confirmed; ^7^ Amplification efficiency (E) and r^2^ in parenthesis determined by dilution method.

## Supplementary Materials

The following are available online at http://www.mdpi.com/2072-6651/10/5/188/s1. Table S1: Sequence results of PCR products and their best hit gene in BLAST.

## Abbreviations

The following abbreviations are used in this manuscript:

ACCA	Acetyl-CoA carboxylase
ACSL1	Acyl-CoA synthetase long-chain family member 1
BDH2	3-hydroxybutyrate dehydrogenase, type 2
BHB	Beta-hydroxybutyrate
BLAST	Basic Local Alignment Search Tool
BW	Body weight
CON	Control group
CON30 or CON60	Control group with 30% or 60% concentrate proportion in the diet
CPT1A	Carnitine palmitoyltransferase 1A
DGAT1	Diacylglycerol O-acyltransferase homolog 1
DIM	Days in milk
DM	Dry matter
DMI	Dry matter intake
DON	Deoxynivalenol
DOM-1	De-epoxy-deoxynivalenol
FASN	Fatty acid synthase
FBP1	Fructose-1,6-bisphosphatase 1
FoxO1	Forkhead box O1
G6Pase	Glucose-6-phosphatase
GPAM	Mitochondrial glycerol phosphate acyltransferase
GUSB	β-glucuronidase
HMGL	3-hydroxymethyl-3-methylglutaryl-CoA lyase
HMGS2	3-hydroxy-3-methylglutaryl-coa synthase 2
IL	Interleukin
IR	Insulin receptor
IRA and IRB	Insulin receptor isoform A and B
LSM	Least square means
MRPS15	Mitochondrial ribosomal protein S15
MYC	Mycotoxin group
MYC30 or MYC60	Mycotoxin group with 30% or 60% concentrate proportion in the diet
NEFA	Non-esterified fatty acid
PC	Pyruvate carboxylase
PCCA	Propionyl-CoA carboxylase, alpha polypeptide
PCK1 and PCK2	Phosphoenolpyruvate carboxykinase 1 and 2
Real-time qPCR	Real-time quantitative polymerase chain reaction
RQUICKI	Revised quantitative insulin sensitivity check index
RPS9	Ribosomal protein S9
SCFA	Short chain fatty acid
SLC2A1, SLC2A2, and SLC2A4	Glucose transporter 1, 2, and 4
SREBP1c	Sterol response element binding protein
TNFa	Tumor necrosis factor alpha
UXT	Ubiquitously-expressed transcript
ZEN	Zearalenone
