# Supplementary material for: Chronic Effects of Fusarium Mycotoxins in Rations with or without Increased Concentrate Proportion on the Insulin Sensitivity in Lactating Dairy Cows
Source: Toxins (Basel). 2018 May 8;10(5):188. doi: 10.3390/toxins10050188 (PMC5983244; doi:10.3390/toxins10050188)
Supplement: Supplementary file 1 [file toxins-10-00188-s001.pdf]

Supplementary Materials: Chronic Effects of *Fusarium* Mycotoxins in Rations with or without Increased Concentrate Proportion on the Insulin Sensitivity in Lactating Dairy Cows

Asako Kinoshita, Christina Keese, Ulrich Meyer, Alexander Starke, Christine Wrenzycki, Sven Dänicke and Jürgen Rehage

Table S1. Sequence results of PCR products and their best hit gene in BLAST.

Symbol	Sequence results of PCR products	BLAST best hit gene (NCBI) description, Sequence ID	Identities
SLC2A1	CGCTCAGCCGCCTACCGCCAGCCCATCCTCATTGCCGTGGTGCTGCAG CTGTCCCAGCAGCTGTCCGGCATCAACGCTGTTTTCTATTACTCCACAA GCATCTTCGAGAAGGCGGGGGTGCAGCAGCCCGTGTATGCCACCATCG GCTCTGGCATCGTCAACACAGCCTTCACTGTCGTGTCTCTA	Bos taurus solute carrier family 2 member 1 (SLC2A1), mRNA NM_174602.2	185/186
SLC2A4	CTGCTGAGATCTGGTCAACGTCCTGCCACGGGTTTCAGGCACTTTTAA GAAGGTGAAGATGAAGAAGCCAAGCAGGAGGACCGCGAATAGAAGA AAGACGTAGGGACCCATAGCATCCGCCACATACTGGAAACCCATGCC GATGATGAAGTTGCATGTCCAGTTGGAG	Bos taurus solute carrier family 2 member 4 (SLC2A4), mRNA NM_174604.1	166/168
FBP1	ACAGAAGATTTCGAAAGATGACCCTTCTGAGAAGGACGCTCTGCAGCC AGGCCGGAACCTGGTGGCTGCTGGCTATGCGCTCTATGGCAGTGCCAC TATGTTGGTCCTGGCCATGGCGAATGGAGTCAACTGCTTCATGCTGGA CCCGGCCATTGGAGAGTTCATTTTGGTGGACAGGGA	Bos taurus fructose-bisphosphatase 1 (FBP1), mRNA NM_001034447.2	176/176
PCK1	GCAGCCGGCTGGCCAGGAGGAGGGGTGGCTGGCAGAGCACATGCTGA TTCTGGGCATCACCAACCCCAAGGGCCAGAAGAAGTACTTCGCGGCTG CGTTTCCCAGCGCCTGTGGGAAGACCAACCTGGCCATGATGAACCCTA CTCTCCCGGGATGGAAAGTAGAGTGTGTGGGTGATGATATCGCCTGGA TGAAATTTGACCAACAAGGTAACTTGCGG	Bos taurus phosphoenolpyruvate carboxykinase 1 (PCK1), mRNA NM_174737.2	219/220
PCK2	CCTGGTATATGAGGCCTTCAACTGGCGCCACGGGGTGTTTGTGGGCAG TGCCATGCGCTCCGAGGCCACTGCAGCGGCTGAACACAAAGGGAAGG TCATCATGCAAGTGGCTA	Bos taurus phosphoenolpyruvate carboxykinase 2, mitochondrial (PCK2), mRNA	105/105

		NM_001205594.1	
PC	TACTGGGGAGGAGCCACCTTTGACGTCGCCATGCGCTTCCTGTACGAG<br>TGCCCCTGGCGACGGCTGCAGGAGCTCCGGGAGCTCGTCCCCAACATC<br>CCATTCCAGATGCTGCTGCGGGGGGCCAACGCCGTGGGCTACACCAAC<br>TACCCCGACAATGTGGTCTTCAAGTTCTGCGAGGTGGCCAAGGAGAAT<br>GGCATGGACATC	Bos taurus pyruvate carboxylase (PC), mRNA NM_177946.4	203/203
CPT1A	GAAGCAGCGTTCTTCGTGACGTTAGACGAAACCGAGCAGGGATACAG<br>GGAGGAGGACCCGGAAACGTCGATGGACAGCTACGCCAAGTCCCTGC<br>TGCATGGCAGGTGTTTCGACAGGTGGTTTGAATGGCTGCCT	Bos taurus carnitine palmitoyltransferase 1A (CPT1A), mRNA NM_001304989.1	124/124
HMGS2	TTCACGCCTTTCTGCAGTTAGTCCAGAAATCCCTGGCCCGCCTGATGTT<br>CAATGACTTCCTGTTGGCCAGTGGTGACACACAGACTGGCATCTACAA<br>GGGCTTGGAGGCCTTCAGGGGACTAAAGCTGGAAAAGAACGCA	Bos taurus 3-hydroxy-3-methylglutaryl-CoA synthase 2 (HMGCS2), mRNA NM_001045883.1	126
HMGL	ATGCTGAGTCTTGAAGGCATCCAGAAATTTCCTGGCGTCAACTACCCA<br>GTCCTGACCCCAAACTTCAAAGGCTTCCAGGCAGCGGTTGCTGCCGGA<br>GCCAAGGAAGTGGCCATCTTTGGAGCCGCCTCTGAACTCTTCACCAAG<br>AAGAACATCAACTGCTCCATAGATGAGAGTTTGCAGCGGTGAGAA	Bos taurus 3-hydroxymethyl-3-methylglutaryl-CoA lyase (HMGCL), mRNA NM_001075132.1	184/186
BDH2	TGTCCATCTCTGCAGAAGATACAAGCCAGACCAAATCCTGAAGAGGC<br>ACTGAGCGATTTCCTAAAGAGACAGAAAACAGGAAGATTTGCAACTG<br>CAGAAGAAGTAGCCCTGCTCTGCGTGTACTTGGCCTCTGATGAATCTG<br>CCTACATCACGGGGAATCCTGTT	Bos taurus 3-hydroxybutyrate dehydrogenase 2 (BDH2), mRNA NM_001034488.2	161/164
ACCA	TACAGTCACTATGAAGTGGATCAGAGATTTCATAGAGAATTTCCTAAA<br>TTTTTCACGTTCCGAGCAAGGGATAAGTTTGAGGAAGATCGTATCTAT<br>CGTCACCTGGAGCCTGCCCTAGCTTTCCAGTTAGAGCTGAACCGGATG<br>AGAAAACTGAA	Bos taurus acetyl-CoA carboxylase alpha (ACACA), mRNA NM_174224.2	146/147

FASN	AGGTCACTGCAGTCCTTCGATGCATCAGGGAATGGC TACTGCCGTGCA GAGGCTGTGATGGCCATCCTTCTGACCAAGAAGTCC CTGGCCCGACGG GTGTACGCCACCATCCTCAACGCTGGCACCAACACG GATGGCTGCAAA GAGAAAGA	PREDICTED: Bos taurus fatty acid synthase (FASN), transcript variant X1, mRNA XM_005220997.2	150/153
GPAM	CTATGGGCATATAGTTGACTACTGCGACAGCAGCAG TTCTTGGAGATT TTTCTGGAAGGCACACGCTCAAGAAGTGGAAAAATC CCTGTGCTCGGG CAGGACTTCTGTCAGTTGTGGTAGATACGCTGTCCAC CAATACGAACT G	Bos taurus glycerol-3-phosphate acyltransferase, mitochondrial (GPAM), mRNA NM_001012282.1	136/141
DGAT1	AGAGCGCCTGGCCGTGGGAGCTCTGACGGAGCAGG CGGGGCTGCTGC TGCACGGGGTCAACCTGGCCACCATTCTCTGCTTCCC AGCGGCCGTGG CCTTTCTCCTCGAGTCTATCACTCCAGTGGGCTCCGT GCTGGCCCTGAT GGTCTACACCATCCTCTTCCTCAAGCTGTTCTCCTAC CGGACCCTCCTG A	Bos taurus diacylglycerol O-acyltransferase 1 (DGAT1), mRNA NM_174693.2	182/182
ACSL1	TGTGTAGTGAGCGATTGTTCAGCATTTGTGAAATGAC AGAGAACACGT TCATTCCTACCTCAGATGACACTTTGATCTCTTTCTTG CCTCTGGCCCA TATGTTTGAGAGAGTTGTAGAGTGTGTGATGCTCTGT CATGGAGCTAA AATAGGATTTTTCCAAGGAGATATCAGGCTGCTTATG GATGATCTCAA GGCCAA	Bos taurus acyl-CoA synthetase long chain family member 1 (ACSL1), mRNA NM_001076085.1	195/196
GUSB	ATTGAGGGTTTCACGAGGATCCACCACTGATGTTCA GTGAAGAGTACC AGAAAGGCCTGCTCCAGCAGTATCATGTGGTTCTGG ACCAAAAACGCA AAGAATATGTGGTTGGCGAGCTCAC	Bos taurus glucuronidase beta (GUSB), mRNA NM_001083436.1	118/119
